# How is the most severe health state being valued by the general population?

**DOI:** 10.1186/s12955-014-0161-9

**Published:** 2014-10-25

**Authors:** Mihir Gandhi, Julian Thumboo, Hwee-Lin Wee, Nan Luo, Yin-Bun Cheung

**Affiliations:** Centre for Quantitative Medicine, Office of Clinical Sciences, Duke-NUS Graduate Medical School, Singapore, Singapore; Biostatistics, Singapore Clinical Research Institute, Singapore, Singapore; Department of International Health, School of Medicine, University of Tampere, Tampere, Finland; Department of Rheumatology and Immunology, Singapore General Hospital, Singapore, Singapore; Department of Medicine, Yong Loo Lin School of Medicine, National University of Singapore, Singapore, Singapore; Department of Pharmacy, Faculty of Science, National University of Singapore, Singapore, Singapore; Saw Swee Hock School of Public Health, National University of Singapore, Singapore, Singapore

**Keywords:** EQ-5D, SF-6D, Utility, Visual analogue scale, Worse than dead

## Abstract

**Background:**

It has been reported that valuation of health states that are close to death, such as the most severe health state, can be affected by health state valuation procedure, and their utility values are difficult to predict. We examined how the most severe health states of Short Form-6 dimension (SF-6D) and EuroQoL-5 dimension-3 level (EQ-5D-3L) were valued by the Singapore general population.

**Methods:**

Overall, 249 SF-6D and 42 EQ-5D-3L states were valued by two separate samples from the Singapore general population using the visual analogue scale (VAS) method. Ordinary least-square regression model was employed to explain deficit in the valuation of the most severe state using the health state descriptors.

**Results:**

A total of 1021 participants from the SF-6D sample and 1015 participants from the EQ-5D-3L sample were included in the analysis. We observed that 67% of the SF-6D participants and 74% of the EQ-5D-3L participants considered the most severe state worse than dead. The most severe state had mean VAS valuation scores more than 20–25 points lower than the adjacent states that are better by only one level in only one dimension. SF-6D VAS valuation score for the most severe state was 27 points and 12 points lower than expected according to the health state descriptors among the participants who considered the most severe state worse than dead and better than dead, respectively. Similar results were found for the EQ-5D-3L valuation.

**Conclusions:**

The most severe health state was valued lower than expected according to its descriptors.

## Background

There is an increasing demand on evaluating the outcome of health-care interventions using cost utility analysis (CUA), and various health regulatory bodies consider it the main approach for evaluating the outcome of health-care interventions [1]. CUA involves quality adjusted life years (QALYs), which are estimated as the time spent in a health state multiplied by its utility. QALYs are very useful as they capture changes in both quality and quantity of life. Health states from generic health outcome instruments such as EuroQoL-5 dimension (EQ-5D), Short Form-6 dimension (SF-6D) and Health Utility Index Mark 3 (HUI3) are valued by the general population using one or more valuation methods such as time trade-off (TTO), standard gamble (SG) and visual analogue scale (VAS) [2]. A number of health states with a mixture of severity levels, including perfect health, dead and the most severe health state described by the instrument, are valued by each participant. The utility of perfect health is valued 1 and dead is valued 0. A negative utility value represents that the health state is considered ‘worse than dead’.

The most severe health state is an important health state in valuation studies. Usually either the most severe health state or dead state is the least valued state and hence decides the lower bound of utility values [3]. For example, the valuation studies in several European countries asked participants to value the most severe health state along with the perfect health and dead state twice because these states are anchoring states (lower or upper bound of the utility values) [4]. Some valuation methods, such as chained TTO and chained SG, also use the most severe health state as the ‘temporary health state’ and ask participants to trade-off or gamble the other health states between the perfect health and the most severe health state [5].

The most severe health state is often labelled as ‘all-worst’ or ‘pits’ [6-10]. There is a general consensus that adding a disease label in disease-specific health state descriptions can have an impact on health state value, possibly due to prior knowledge or preconception of the disease [11]. However, no study to our knowledge has investigated the impact of labeling a generic health state. Valuation of health states based on generic instruments is the most common method for eliciting the population preferences in CUA, thus how the most severe health state (in the presence of a label) is valued needs attention.

Drawing on data from a valuation study of the two most commonly used generic instruments, namely SF-6D and EQ-5D-3 level (EQ-5D-3L), we aim to examine how the most severe health state is valued by the participants. In this study, the most severe health state was labelled as ‘all-worst’ state. A valuation study in multi-ethnic Asian general population showed that the majority of participants felt that ‘all-worst’ is a better description than ‘pits’ for the most severe health state of EQ-5D [12].

## Methods

### Valuation survey procedures

A cross-sectional, face-to-face survey of health state valuation for SF-6D and EQ-5D-3L using the VAS method was conducted in 2009 from a representative sample of the general population of Singapore, a multi-ethnic Asian country. A multi-stage sampling approach was used to randomly select residential blocks, within which households were selected. Potential participants who satisfied the pre-set recruitment quotas for ethnicity (400 Chinese, 400 Malay, 234 Indian), gender (50% Female) and age (30% for 21–34 years, 40% for 35–49 years, 30% for 50+ years) were interviewed. Within each race, there was a quota that half of the participants would use English and the remaining half would use their native language for the interviews, i.e. Mandarin for Chinese, Malay for Malays and Tamil for Indians.

Two separate samples of 1034 participants each were selected for the SF-6D and EQ-5D-3L health states valuation. The SF-6D consists of 6 dimensions (physical functioning, role limitations, social functioning, pain, mental health and vitality) and each dimension has 4 to 6 levels. Thus, SF-6D describes a total of 18,000 health states. A subset of 249 SF-6D states was selected (out of 18,000) for the valuation based on the protocol of Brazier *et al*. [6]. The most severe state (‘645655’ for SF-6D) was labeled as ‘all-worst’. Each participant was asked to compare between ‘dying now’ and ‘living for the rest of his/her life in all-worst’, from which the less desirable state was assigned a value 0 on the VAS. Each participant was then asked to value a unique set of 6 states from the subset of SF-6D states and either dead or the ‘all-worst’ state, depending on which one was not valued earlier at 0. The unique set of 6 health states were assigned to each participant in a way that they spread widely over the valuation space. A 100-point “feeling thermometer” with endpoints of 100 (most desirable, i.e. perfect health) and 0 (least desirable) was used as the VAS. The participants were required to indicate where they would rate each of the assigned states on the “feeling thermometer” by imagine themselves in that state for the rest of their life without changing. The participants were allowed to value more than one health state at the same level of VAS.

The valuation of EQ-5D-3L states was carried out in a similar way as SF-6D. The EQ-5D-3L consists of 5 dimensions (mobility, self-care, usual activities, pain/discomfort, and anxiety/depression) with 3 levels each (no problems, some problems, and extreme problems) and thus describes 243 health states. A subset of 42 EQ-5D-3L states was selected based on the protocol of Dolan [13]. The most severe state (‘33333’) was labeled as ‘all-worst’. Unconscious state was also valued in addition to the other 6 assigned states.

The study was approved by SingHealth Centralized Institutional Review Board.

### Analyses

Participants who met the following criteria were excluded from our analysis: a) valued less than 3 health states, b) did not value dead or the ‘all-worst’ state, c) valued dead or the ‘all-worst’ state or unconscious state higher than all the other states, d) gave the same valuation score to all the health states, e) self-reported or rated by the interviewers as having poor understanding of health states description or valuation tasks. The valuation score used in the analyses was ‘raw’ VAS valuation score ranging from 0 (worst possible score) to 100 (best possible score). We did not transform the valuation scores to utility to avoid an impact of the transformation on the estimated valuation score [3].

Mean valuation scores of the health states are presented using line graphs for a subset of selected health states. As the study has valued many health states, to maintain visual clarity we did not include all the valued health states. The lowest ten health states with the least valuation scores near the dead state were included in the graphs. Health states with higher valuation scores were systematically skipped for the graphical presentation. The valuation scores among the participants who considered the ‘all-worst’ state worse than dead and who considered the ‘all-worst’ state better than dead were presented separately in the graphs.

We performed ordinary least-square (OLS) regression model for EQ-5D-3L with valuation score as the dependent variable and indicator variables representing level of severity for each dimension as the independent variables, with an intercept [4]. That is, included 2 indicator variables for each of the 5 dimensions of EQ-5D-3L. We also included an indicator variable (N_3_) to take into account of additional disutilities when severe problem (level 3) is reported on at least one dimension. In addition to the above commonly used variables, we included two indicator variables D_1_ and D_2_ and their interaction: D_1_ represented the participant who considered the ‘all-worst’ state worse than dead and D_2_ represented the ‘all-worst’ state. This model helped to assess whether there was a deficit in the valuation score for the most severe state even after taking the descriptors (levels and dimensions) into account. It also assessed possible impact of considering the most severe state worse than death on its valuation.

Similar regression analysis model was used to study SF-6D valuation scores. For the variable N_3_, the severe level was defined as levels 4–6 for physical functioning, levels 3–4 for role limitation, level 4–5 for social functioning, mental health and vitality, and level 5–6 for pain [6].

All the health states valued in the study were included in the regression analysis. Perfect health state was not included in the models, as it was assigned a value 100 on VAS. The dead and unconscious states were also excluded from the regression analyses as they do not represent any health states/dimensions of SF-6D or EQ-5D-3L. Since each of the participants valued 6 health states, we used the Eicker-Huber-White robust standard error for clustered data for statistical inference [14]. A P-value less than 0.05 was considered statistically significant. All the analyses were carried out using Stata/MP 10.1 for Windows.

## Results

Demographic and health characteristics information was received from all 1034 participants of the SF-6D sample. Seven participants valued dead higher than all the other states; 1 participant valued the ‘all-worst’ state higher than all the other states; and 5 participants were observed to have poor understanding of heath states description and/or valuation tasks. Hence, these 13 participants were excluded from the SF-6D related analysis. Table 1 shows demographic and health characteristics of 1021 participants for the SF-6D valuation that were included in the analysis. Due to the pre-specified quota for gender, age and ethnicity, the demographic characteristics of enrolled participants were similar to what was planned. The majority of the SF-6D participants were married (n = 765, 75%), employed/self-employed (n = 659, 65%), had at least secondary education (n = 844, 83%), and self-reported good to excellent general health (n = 947, 93%).

**Table 1:** **Demographic and health characteristics of the study sample participants**

**Characteristics, n (%)**	**Participants completing the SF-6D**	**Participants completing the EQ-5D-3L**
	**(N = 1021)**	**(N = 1015)**
Female	521 (51.0)	512 (50.4)
Age (years)		
21-29	190 (18.6)	194 (19.1)
30-39	222 (21.7)	228 (22.5)
40-49	272 (26.6)	269 (26.5)
50-59	206 (20.2)	200 (19.7)
60+	131 (12.8)	124 (12.2)
Ethnicity		
Chinese	392 (38.4)	387 (38.1)
Malay	396 (38.8)	399 (39.3)
Indian	233 (22.8)	229 (22.6)
Education level		
Primary (6 years) or less	177 (17.3)	190 (18.7)
Secondary (11 years)	562 (55.0)	576 (56.8)
Diploma/degree or higher	282 (27.6)	249 (24.5)
Married/partner	765 (74.9)	761 (75.0)
Employed or self-employed	659 (64.5)	643 (63.4)
General health status		
Poor	4 (0.4)	8 (0.8)
Fair	70 (6.9)	99 (9.8)
Good	443 (43.4)	424 (41.8)
Very good	419 (41.0)	383 (37.7)
Excellent	85 (8.3)	101 (10.0)
Self-reported health on EQ-5D-3L VAS, Mean (SD)	84.5 (11.2)	83.0 (12.1)

VAS = visual analogue scale.

Similarly in the EQ-5D-3L sample, 12 participants valued dead higher than all the other states; 2 participants valued the unconscious state higher than all the other states; 1 participant did not value the ‘all-worst’ state; and 4 participants were observed to have poor understanding of heath states description and/or valuation tasks. Hence, 19 participants were excluded from the EQ-5D-3L related analysis. The demographic and health characteristics of the EQ-5D-3L participants were similar to the SF-6D participants (Table 1).

**Figure 1:**
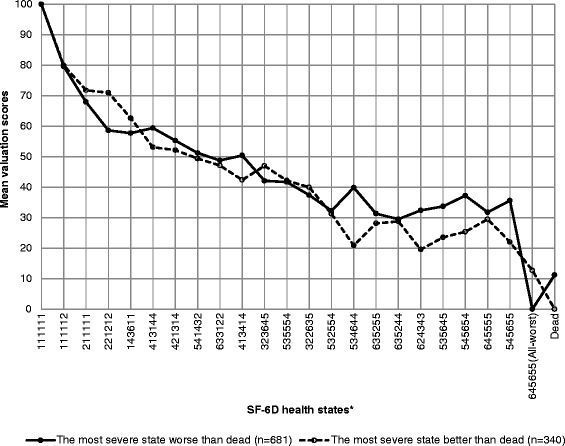
**Mean valuation scores of SF-6D health states.** *The lowest ten health states with the least valuation scores near the dead state are included in the graphs. Health states with higher valuation scores were systematically skipped for the graphical presentation.

In the SF-6D valuation, except the ‘all-worst’ state, no other health state was valued worse than dead state. The majority of participants (n = 681, 67%) considered the ‘all-worst’ state worse than dead. The mean valuation score given to the ‘all-worst’ state was 12.8 (SD = 14.0) among the participants who considered the ‘all-worst’ state better than dead. On the other hand, dead state was valued with mean valuation score of 11.2 (SD = 12.5) among the participants who considered the ‘all-worst’ state worse than dead. The mean valuation scores for selected SF-6D health states are shown in Figure 1. For the participants who considered the ‘all-worst’ state worse than dead, there was a difference of more than 30 points in the mean valuation score between the ‘all-worst’ state (‘645655’) and its adjacent health states that are only one level different in one dimension (‘545655’ and ‘645555’). For the participants who considered the ‘all-worst’ state better than dead the corresponding difference ranged from 9 to 17 points.

**Figure 2:**
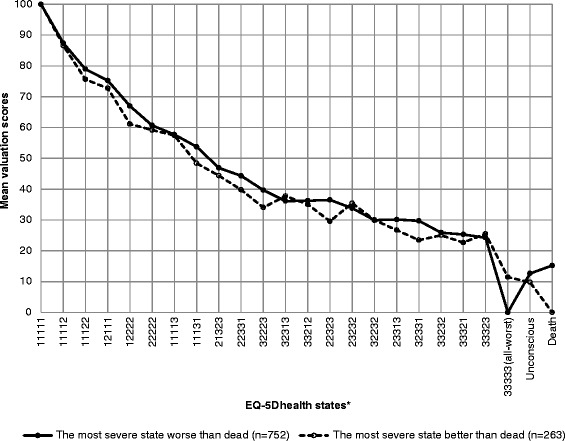
**Mean valuation scores of EQ-5D-3L health states.** *The lowest ten health states with the least valuation scores near the dead state are included in the graphs. Health states with higher valuation scores were systematically skipped for the graphical presentation.

Similar to the SF-6D results, only the ‘all-worst’ state was valued worse than dead, and the majority of participants (n = 753, 74%) considered the ‘all-worst’ state worse than dead in the EQ-5D-3L valuation. The mean valuation score of the ‘all-worst’ state was 11.4 (SD = 12.3) among the participants who considered the ‘all-worst’ state better than dead; and the mean valuation score of dead was 15.2 (SD = 19.7) among the participants who considered the ‘all-worst’ state worse than dead. Similar to Figures 1, 2 also shows a difference of 25 points in the mean valuation score between the ‘all-worst’ state (‘33333’) and its adjacent state ‘33323’ for the participants who considered the ‘all-worst’ worse than dead. For the participants who considered the ‘all-worst’ better than dead the corresponding difference was 11 points.

The regression intercept showed that, among the participants who considered the ‘all-worst’ state better than dead in the SF-6D sample, the estimated valuation score was 80.2 (95% CI: 77.3, 83.1) when all the dimensions were at its best (level 1) (Table 2). The coefficient D_1_ showed that the participants who considered the ‘all-worst’ state worse than dead scored the other health states higher by 2 points (95% CI: 0.2, 3.8) compared to the participants who considered the ‘all-worst’ state better than dead. The participants who considered the ‘all-worst’ state better than dead scored the ‘all-worst’ state 12.3 points lower (95% CI: −15.8, −8.8; P-value < 0.001) than expected by its descriptors. Furthermore, the participants who considered the ‘all-worst’ state worse dead scored the ‘all-worst’ state 27.0 points lower (coefficient: −12.3-14.7 = 27.0; 95% CI: −30.2, −24.0; P-value < 0.001) than expected.

**Table 2:** **Summary of ordinary least-square regression on valuation score of SF-6D health states**

Regressor*	**Coefficient**	**95% confidence interval**	**P-value**
The most severe state worse than dead (D_1_)	2.0	[0.2, 3.8]	0.029
The most severe state (D_2_)	−12.3	[−15.8, −8.8]	<0.001
Interaction of D_1_ and D_2_	−14.7	[−16.6, −12.9]	<0.001
At least one severe level (N_3_)	−2.3	[−4.3, −0.4]	0.018
Physical functioning level 2	−7.5	[−16.6, −12.9]	<0.001
Physical functioning level 3	−7.9	[−9.7, −6.0]	<0.001
Physical functioning level 4	−13.8	[−15.8, −11.7]	<0.001
Physical functioning level 5	−13.9	[−15.9, −11.9]	<0.001
Physical functioning level 6	−22.1	[−24.4, −19.9]	<0.001
Role limitations level 2	−3.7	[−5.2, −2.2]	<0.001
Role limitations level 3	−3.1	[−4.6, −1.6]	<0.001
Role limitations level 4	−2.7	[−4.3, −1.0]	0.002
Social functioning level 2	−3.1	[−4.6, −1.5]	<0.001
Social functioning level 3	−2.1	[−3.8, −0.4]	0.015
Social functioning level 4	−2.9	[−4.8, −1.1]	0.002
Social functioning level 5	−3.2	[−5.1, −1.4]	0.001
Pain level 2	−3.9	[−5.7, −2.2]	<0.001
Pain level 3	−6.3	[−8.1, −4.5]	<0.001
Pain level 4	−6.8	[−8.6, −4.9]	<0.001
Pain level 5	−7.5	[−9.2, −5.7]	<0.001
Pain level 6	−11.1	[−13.0, −9.2]	<0.001
Mental health level 2	−2.1	[−3.8, −0.4]	0.016
Mental health level 3	−2.8	[−4.7, −1.0]	0.002
Mental health level 4	−4.0	[−5.9, −2.1]	<0.001
Mental health level 5	−3.0	[−4.9, −1.1]	0.002
Vitality level 2	−0.3	[−2.1, 1.6]	0.785
Vitality level 3	−5.0	[−6.9, −3.2]	<0.001
Vitality level 4	−5.6	[−7.6, −3.6]	<0.001
Vitality level 5	−10.6	[−12.6, −8.6]	<0.001
Intercept	80.2	[77.3, 83.1]	<0.001
R^2^ = 0.519			

*Adjusted for all levels of 6 dimensions of SF-6D.

For the EQ-5D-3L valuation, the participants who considered the ‘all-worst’ state better than dead scored the ‘all-worst’ state 2.1 points lower (95% CI: −5.4, −1.1; P-value = 0.201) than expected by its descriptors. The participants who considered the ‘all-worst’ state worse than dead scored the ‘all-worst’ state 15.8 points lower (coefficient = −2.1-13.7 = −15.8; 95% CI: −18.8, −12.7; P-value < 0.001) than expected (Table 3).

**Table 3:** **Summary of ordinary least-square regression on valuation score of EQ-5D-3L health states**

Regressor*	**Coefficient**	**95% confidence interval**	**P-value**
The most severe state worse than dead (D_1_)	2.2	[−0.0, 4.5]	0.054
The most severe state (D_2_)	−2.1	[−5.4, 1.1]	0.201
Interaction of D_1_ and D_2_	−13.7	[−15.8, −11.6]	<0.001
At least one severe level (N_3_)	−14.8	[−17.4, −12.1]	<0.001
Mobility level 2	−8.2	[−9.4, −6.9]	<0.001
Mobility level 3	−15.0	[−17.0, −13.0]	<0.001
Self-care level 2	−5.1	[−6.5, −3.7]	<0.001
Self-care level 3	−13.9	[−15.7, −12.2]	<0.001
Usual activities level 2	−1.6	[−3.5, 0.3]	0.102
Usual activities level 3	−4.2	[−6.3, −2.0]	<0.002
Pain/discomfort level 2	−5.1	[−6.3, −3.9]	<0.001
Pain/discomfort level 3	−12.4	[−13.6, −11.1]	<0.001
Anxiety/depression level 2	−0.3	[−1.8, 1.2]	0.674
Anxiety/depression level 3	−6.1	[−7.9, −4.2]	<0.001
Intercept	79.9	[77.5, 82.3]	<0.001
R^2^ = 0.642			

*Adjusted for all levels of 5 dimensions.

All the coefficients of indicator variables for different levels of SF-6D and EQ-5D-3L dimensions in the respective regression models were negative and most of them were statistically significant (Tables 2 and 3).

## Discussion

We examined the valuation of the most severe state (labeled as ‘all-worst’) of two of the most widely used generic instruments, SF-6D and EQ-5D-3L, using the VAS method in a general population. We examined the valuation score in two groups of participants - who considered the most severe state worse than dead and who considered it better than dead. We observed a deficit of 20–25 points in the valuation score of the most severe state than the adjacent states that are better by only one level in only one dimension. The regression analysis showed that this deficit could not be explained by the differences in the health state descriptors. In the SF-6D valuation, the unexplained deficit was about 27 points and 12.3 points, respectively, among participants who considered the most severe state worse and better than dead. This is practically significant, as 3.3 points was considered minimally important difference [15,16]. In the valuation of the EQ-5D-3L, the participants who considered the most severe state worse than dead also scored the most severe state 15.8 points lower than expected according to its descriptor, which is bigger than the minimally important difference of 7–8 point [16,17]. The regression models were developed based on the comparative review and user guide for EQ-5D value sets [4]. We used raw VAS valuation score in the analysis without any transformations or rescaling to reduce artifact effect on the regression coefficients [3].

We found a deficit in value in the health state labelled as ‘all-worst’. Participants may have valued the most severe state based on the ‘all-worst’ label rather than the objective description of it [18]. That is, the valuation might be affected by prior belief associated with a worst health condition or an emotional response to the hearing of ‘all-worst’. Several studies of disease-specific health states valuation have found that an inclusion of disease label lowered the health state values [11]. For example, a study found that using a label ‘breast cancer’ reduced health state values [19]. Another study found that the use of mental health labels such as mental handicap, schizophrenia and dementia was associated with lower health state values [20]. The present study showed a similar labeling effect in a generic health state and in a community context. Thus, we suggest to avoid labeling health states in valuation studies.

Furthermore, the difference in the degree of deficit in the values of the most severe state between participants who considered the state worse than dead and those who considered it better than dead might have been affected by end-aversion bias [21]. Some participants might be reluctant to value health states at the extreme end of the VAS scale or the portion of scale near the end. For the participants who consider the most severe health state worse than dead, the valuation procedure involved putting the card that represented the state at the lower end of the VAS scale (score 0). Then, the remaining health states were valued between the two ends of the VAS scale. Thus, the other health states near the lower end of the VAS scale might have been valued higher than the most severe health state as a result of end-aversion bias. On the other hand, for the participants who considered the most severe health state better than dead, the dead state was anchored at 0 and the most severe health state and the other health states were valued similarly between the two ends of the VAS scale.

Our study showed that there was larger unexplained deficit in the valuation of the most severe state in SF-6D than EQ-5D-3L. It was likely due to differences in their descriptive systems. It has been shown by Brazier *et al.* [22] that severe SF-6D states are less severe than the severe EQ-5D-3L states. That is, the participants consider the most severe state of EQ-5D-3L almost like the worst state based on its descriptors and hence leave less room for the other factors which can intensify its severity, whereas in SF-6D there is more scope for other factors, such as the labeling effect.

It should be noted that we valued the health states using the VAS method and hence the study findings are applicable to this valuation method only. Further research would be needed to examine if the present findings are generalizable to other valuation methods like TTO and SG.

## Conclusions

We found that the most severe state was valued significantly lower than expected according to its descriptors. The magnitude of the deficit depended on the valuation instrument and whether the respondents considered the most severe state worse or better than dead.
